# The Implementation of China's Mental Health Law-Defined Risk Criteria for Involuntary Admission: A National Cross-Sectional Study of Involuntarily Hospitalized Patients

**DOI:** 10.3389/fpsyt.2018.00560

**Published:** 2018-11-06

**Authors:** Feng Jiang, Huixuan Zhou, Jeffrey J. Rakofsky, Linlin Hu, Tingfang Liu, Huanzhong Liu, Yuanli Liu, Yi-lang Tang

**Affiliations:** ^1^School of Public Health, Chinese Academy of Medical Sciences and Peking Union Medical College, Beijing, China; ^2^Department of Psychiatry and Behavioral Sciences, Emory University, Atlanta, GA, United States; ^3^Institute for Hospital Management, Tsinghua University, Beijing, China; ^4^Department of Psychiatry, Chaohu Hospital of Anhui Medical University, Hefei, China; ^5^Atlanta VA Medical Center, Decatur, GA, United States

**Keywords:** mental health law, risk criteria, implementation, psychiatric hospital, involuntary admission

## Abstract

**Objective:** Involuntary admission is one of the most controversial issues in psychiatry in China. This study aimed to examine the implementation of the new risk criteria for involuntary admission, as defined by the new Mental Health Law (MHL), in major psychiatric hospitals; and to explore factors associated with the implementation.

**Method:** We selected 32 psychiatric hospitals in 29 provincial capital cities in mainland China. We included all involuntarily admitted psychiatric inpatients who were discharged from December 25 to 27, 2017. Patients' demographic and clinical data and reasons for admission were retrieved. Hospitals' information was also collected. Multilevel logistic regression was applied to explore factors associated with the implementation.

**Results:** We collected valid data from 814 inpatients. Rates of risk criteria implementation ranged from 7.9 to 88.5% in these hospitals. Only 369 inpatients (45.3%) met the MHL-defined risk criteria. Overall, between 62.2 and 78.5% of the variance in risk criteria implementation was at the patient level, and between 21.5 and 37.8% of the variance was at the hospital level. Patients with higher Global Assessment of Functioning (GAF) scores at admission were less likely to meet the risk criteria (OR 1.02, 95% CI 1.01–1.03). No statistically significant association was found between risk criteria implementation and other patient level or hospital level factors.

**Conclusion:** Our findings show the implementation rate of the MHL's risk criteria overall was low, with only 45.3% of involuntary admissions meeting the MHL-defined criteria. This suggests that some patients' civil rights might have been violated.

## Introduction

After 27 years of contentious debate, China's first Mental Health Law (MHL) was passed and implemented in 2013. The MHL is comprehensive and addresses many aspects of mental health services. One of its components aims to provide a legal foundation to protect patients' rights and to guide involuntary admission for patients who are at high risk of harming themselves or others (1). This largely followed the examples of laws passed in the United States (2), but it did not include the category involving individuals who present a life-endangering crisis because of their inability to care for themselves. In the MHL, the criteria for involuntary admission are based primarily on risk, not on the need for treatment (3). In other words, only when individuals with severe mental disorders present a risk of harming themselves or others, can involuntary admission be initiated by their family members or psychiatrists.

Since the law took effect 5 years ago, debates over how to interpret and implement the criteria have continued. Some argue the definition of risk in MHL is vague and not operational (4), and psychiatrists in China differ greatly in their attitudes toward the procedure for involuntary psychiatric admissions (5). A survey showed that, after MHL took effect, involuntary admission is still the most common type of admission for patients with psychotic disorders (6). Risk of harm, assessment of need, treatment attitude, and patients' functioning may all have an impact on how the risk criteria are implemented (6, 7). As of now, there have been no published studies focusing on the implementation of risk criteria for involuntary admission in China. This study, based on a national survey, aimed to examine the implementation of the criteria for involuntary admission in major psychiatric hospitals, and to explore potential factors associated with the implementation.

## Methods

This study was a part of a larger research project, the National Survey for the Evaluation of Psychiatric Hospital Performance, which aimed to evaluate the performance of major psychiatric hospitals in China. We selected the provincial psychiatric hospitals under the jurisdiction of the Ministry of Health in each capital city in 29 out of 31 provinces in mainland China (Gansu and Tibet were not included because at the time there were no such hospitals in their capital cities). In total, 32 psychiatric hospitals were selected. One hospital in each capital city of most provinces, except Beijing (3 selected) and Anhui Province (2 selected). We included all involuntarily admitted psychiatric inpatients who were discharged from December 25 to 27, 2017. Patients' demographic and clinical data, including the Global Assessment of Functioning (GAF) scores (8), and reasons for admission were retrieved from the medical records by research staff. The documented reasons for admission in medical records were assessed to determine whether the involuntary admission met the MHL-defined risk criteria. If the admission reasons included an attack on others/themselves or endangering public security or impulsive aggression, the case would be deemed to have met the MHL-defined risk criteria. Hospitals' information was also collected. We excluded patients held for <72 h for assessment only and excluded patients with incomplete data.

Statistical summaries were calculated using the SPSS (version 23). Multilevel logistic regression was applied with MLwiN (version 2.30) to estimate variation in risk criteria implementation. Following convention, we assumed the binary outcome was defined by a continuous latent variable and patient-level variance was standardized to the logistic variance of π2/3 = 3.29 (9). The variance partition coefficient (VPC) was calculated at each level. MCMC Bayesian methods were used to estimate all models. We used the Bayesian Deviance Information Criterion (DIC) statistic to compare the fit of models, which is the lower the better. Odds ratios (ORs; 95% confidence intervals [CI]) are also reported. We do not report *p*-values, in keeping with standard practice for reporting Bayesian model results.

## Results

In all, there were 871 involuntarily admitted inpatients during this study period and valid data was collected from 814 of the involuntarily admitted inpatients. A wide range in rates of implementation was observed in 32 hospitals, ranging from 7.9 to 88.5%.

Among 814 inpatients, 369 inpatients (45.3%) met the MHL-defined risk criteria. Among these 369 patients, 85 were described to have risk of suicide or self-injury, 310 had risk of harming others, and 26 had both. Among the rest of those involuntarily admitted patients, 353 patients were admitted for psychotic symptoms, 222 for mood symptoms, 36 for medication adjustment, 92 for poor self-care ability, and 258 for multiple reasons.

Table 1 shows the characteristics of these involuntarily admitted inpatients.

**Table 1:** Characteristics of 814 involuntary psychiatric inpatients ^a^.

**Characteristic**	**Total sample**		**Met risk criteria**		**Not met risk criteria**		***p***
	***N***	**%**	***N***	**%**	***N***	**%**	
Sex							0.023^*^^b^
Male	410	50.4	202	54.7	208	46.7	
Female	404	49.6	167	45.3	237	53.3	
Education							0.384 ^b^
Elementary school	127	15.9	66	17.9	63	14.2	
Middle school	247	30.3	114	30.9	133	29.9	
High school	236	29.0	105	28.5	131	29.4	
College/university or above	202	24.8	84	22.8	118	26.5	
Marital							0.194 ^b^
Not married (Single and others)	443	54.4	210	56.9	233	52.4	
Married	371	45.6	159	43.1	212	47.6	
Diagnosis							0.670 ^b^
Schizophrenia and related disorders	494	60.7	223	60.4	271	60.9	
Mood disorders	211	25.9	100	27.1	111	24.9	
Others	109	13.4	46	12.5	63	14.2	
Age	40.7 ± 15.4		39.6 ± 15.0		41.6 ± 15.8		0.062^c^
GAF	41.2 ± 17.7		39.2 ± 18.2		42.9 ± 17.1		0.003^*^^c^

*GAF, Global Assessment of Functioning*.

a *Statistical tests were 2-tailed, P < 0.05 was considered significant*.

* *Significant P-values*.

b *Chi-square test*.

c *t-test*.

Overall, between 62.2 and 78.5% of the variance in risk criteria implementation was at the patient level, and between 21.5 and 37.8% of the variance was at the hospital level (Table 2).

**Table 2:** Independent contributors to implementation of the new criteria for involuntary admission under China's Mental Health Law, by two-level logistic model analysis.

	**Null model**	**Model with patient-level covariates**	**Model with patient-level, hospital-level covariates**
**PATIENT LEVEL**			
Sex(ref. female)			
Male		0.67 (0.46–0.98)	0.69 (0.46–1.05)
Education(ref. Elementary school)			
Middle school		1.4 (0.84–2.31)	1.22 (0.67–2.19)
High school		1.49 (0.89–2.5)	1.16 (0.63–2.15)
College/university or above		1.62 (0.91–2.88)	1.07 (0.54–2.13)
Marital(ref. not married)			
Married		1.12 (0.75–1.69)	1.21 (0.82–1.79)
Diagnosis(ref. others)			
Schizophrenia and related disorders		1.13 (0.64–2)	1.27 (0.68–2.39)
Mood disorders		0.78 (0.43–1.41)	0.76 (0.38–1.52)
Age		1.01 (1.00–1.03)	1.01 (0.99–1.02)
GAF		1.02 (1.01–1.03)	1.02 (1.01–1.03)
**HOSPITAL LEVEL**			
Beds			1.00 (1.00–1.00)
Doctors			1.00 (1.00–1.00)
**UNEXPLAINED VARIANCE**			
Patient level variance	3.29	3.29	3.29
Hospital level variance(SE)	2.00 (0.97)	1.32 (0.48)	0.90 (0.60)
**PERCENTAGE OF UNEXPLAINED VARIANCE**			
Patient level	62.19	71.37	78.52
Hospital level(95% CI)	37.81 (2.92–97.08)	28.63 (10.33–40.73)	21.48 (0–38.69)
**DIC**	1107.52	1090.37	1064.72

*DIC, deviance information criterion. p-values not reported in keeping with standard practice for reporting Bayesian model results. The reviewer FH and handling editor declared their shared affiliation at the time of the review*.

At the patient level, after adjusting for all covariates, patients with lower GAF scores at admission were more likely to meet the risk criteria (OR 1.02, 95% CI 1.01–1.03). No statistically significant association was found between risk criteria implementation and other patient level or hospital level factors.

## Discussion

Our findings, based on a national survey, show the implementation rate of the MHL's risk criteria overall was low, with only 45.3% of involuntary admissions meeting the MHL-defined risk criteria. This suggests that some patients' civil rights might have been violated. Notably, a wide range in the rates of involuntary admissions meeting MHL-defined criteria was observed across different hospitals and there were significant variations in risk criteria implementation at the hospital and at the patient level. In addition to the hospital-level variables already included, the variation in implementation across different hospitals may have been influenced by factors not listed in our survey, including: the individual doctor's attitude or interpretation of risk criteria, the local process of involuntary admission, the public attitude to and interpretation of MHL-defined risks, and local socio-cultural factors (5, 10).

In 2015, there were 1650 mental health hospitals in China (11). According to the MHL, all psychiatric hospitals can receive involuntary patients. This survey was conducted in capital cities and the sample size within each hospital was limited as we only included discharged patients in a selected period. Both of these factors can affect the generalizability of these results to other psychiatric inpatients. Further, recall bias and observational bias cannot be ruled out.

Future research is needed to elaborate additional factors that may account for the variation in the MHL risk criteria being properly implemented, and to understand what role these factors play in this process. The more we understand this, the closer we will come to honoring patient autonomy without compromising public safety.

## Ethics statement

The study was reviewed and approved by the Ethics Committee (IRB) at the School of Public Health, Peking Union Medical College. Written informed consent was obtained from all participants.

## Author contributions

FJ, YL and YT had full access to all the data in the study and take responsibility for the integrity of the data and the accuracy of the data analysis. FJ conducted the data analysis. FJ, HZ, LH, TL, HL, and YL were responsible for data acquisition and contributed to its interpretation. JF, JR, and YT wrote the manuscript, and all the co-authors revised its content. All the authors approved the final version of the manuscript and agreed to be held accountable for all aspects of the work.

### Conflict of interest statement

The authors declare that the research was conducted in the absence of any commercial or financial relationships that could be construed as a potential conflict of interest. The reviewer FH and handling editor declared their shared affiliation at the time of the review.
